# Helminth-derived stefin-1 selectively reduces leukemic cell viability and promotes apoptosis in U937 cells

**DOI:** 10.1371/journal.pone.0353364

**Published:** 2026-07-27

**Authors:** Phornthip Ploensil, Yaneenart Suwanwong, Phatchariya Phannasil, Mayuri Tarasuk, Unchalee Thonsri, Pakkawan Prapasitsin, Pongsakorn Martviset, Pathanin Chantree

**Affiliations:** 1 Graduate Program in Clinical Hematology Sciences, Faculty of Allied Health Sciences, Chulalongkorn University, Bangkok, Thailand; 2 Center of Excellence for Biosensors and Bioengineering (CEBB), Chulalongkorn University, Bangkok, Thailand; 3 Thalassemia Research Center, Institute of Molecular Biosciences, Mahidol University, Nakhon Pathom, Thailand; 4 Graduate Program in Bioclinical Sciences, Chulabhorn International College of Medicine, Thammasat University, Pathumthani, Thailand; 5 Department of Preclinical Science, Faculty of Medicine, Thammasat University, Pathumthani, Thailand; 6 Graduate Program in Applied Biosciences, Faculty of Medicine, Thammasat University, Pathumthani, Thailand; 7 Thammasat University Research Unit in Medical Application of Biomolecules, Thammasat University, Pathumthani, Thailand; Duke University Medical Center: Duke University Hospital, UNITED STATES OF AMERICA

## Abstract

Leukemia remains a challenging hematological malignancy, particularly due to treatment resistance and the limited availability of therapies that selectively target cancer cells. Helminth-derived cysteine protease inhibitors (stefins) are mainly known for their roles in host–parasite interactions, but their effects on cancer cells have not been well explored. In this study, we investigated the biological activity of recombinant *Fasciola gigantica* Stefin-1 (rFgStefin-1) in the human U937 leukemic cell line. Treatment with rFgStefin-1 reduced cell viability in a dose- and time-dependent manner, with an IC₅₀ of approximately 11 µM at 48 h. In contrast, normal peripheral blood mononuclear cells (PBMCs) showed minimal changes under the same conditions. Flow cytometry analysis demonstrated increased apoptotic cell populations together with mitochondrial membrane depolarization following rFgStefin-1 treatment. At the molecular level, rFgStefin-1 treatment was associated with an increased BAX/BCL-2 ratio, increased cleaved caspase-3 expression, reduced AKT-associated signaling, and enhanced PARP cleavage. These findings were supported by both qRT-PCR and Western blot analyses. Overall, these results suggest that rFgStefin-1 promotes apoptosis in leukemic cells while exerting limited effects on normal cells under the tested conditions. Further studies are required to clarify the underlying molecular mechanisms and to evaluate the effects of rFgStefin-1 in additional leukemia models and *in vivo* systems.

## Introduction

Leukemia, a blood cancer, is one of the deadliest cancers and the leading cause of cancer deaths worldwide, accounting for approximately 5% of all cancer cases, and ranks sixth among the most aggressive cancers found in humans. It is also responsible for about 30% of cancer-related deaths in children [1]. Leukemia can be classified according to its progression into chronic and acute leukemia. Acute myeloid leukemia (AML) is an invasive cancer characterized by the rapid growth of abnormal white blood cells. Additionally, it is the most prevalent form of cancer brought on by genetic abnormalities, which can impair apoptosis and differentiation. Currently, chemotherapy is the main treatment for leukemia. Although allogeneic hematopoietic stem cell transplantation is therapeutic, some patients are unable to have a transplant due to age factors or a lack of a donor. The current state of chemotherapy in leukemia has not changed in more than 40 years. Despite a good response, approximately 45%–65% of AML patients achieve complete symptom relief, but the rate of drug resistance and recurrence is still high [2]. The pathophysiology of bone marrow contributes to chemoresistance in leukemia cells through cell-cell and cell-matrix interactions [3]. Due to the cytotoxic drug’s generalized toxicity to both malignant and normal cells, the severe side effects of chemotherapy remain a problem. Therefore, the development of novel drugs targeting specific leukemia targets is required. Natural bioactive compounds are of particular interest for anticancer activity. Recently, protease inhibitors have gained significant attention as potential anticancer agents. Proteolytic dysregulation, an imbalance between protease enzymes and their inhibitors, has been associated with multiple stages of tumor growth. Among cysteine proteases, overexpression of cathepsin B and cathepsin L has an important role in leukemogenesis [4]. Cystatins, or stefins, are naturally tight-binding, reversible inhibitors of cysteine proteases found in almost all nucleated cells of all living organisms, from parasites to humans. Previous studies reported that stefin-1 (stefin A) strongly inhibits cathepsin B and L [5–7]. In addition to their role in protease regulation, cystatins have also been linked to cancer-related processes. For example, recombinant human stefin-1 has been reported to suppress cancer cell proliferation by modulating MAPK and AKT signaling pathways [8]. Furthermore, increasing evidence suggests that cathepsins and their endogenous inhibitors contribute to cancer-associated cellular processes, including apoptosis resistance, cell survival, and leukemic progression [9,10]. Dysregulated cathepsin activity has been implicated in the maintenance of malignant phenotypes in leukemia, while cystatin/stefin family proteins have been associated with modulation of apoptosis-related signaling pathways [11]. Therefore, the biological functions of stefin/cystatin proteins provided a rationale for investigating whether helminth-derived rFgStefin-1 could influence leukemic cell viability and apoptosis-associated signaling in leukemic cells.

In parasitic helminths, stefins are primarily studied for their role in host–parasite interactions, particularly in immune modulation and evasion of host responses [12]. A stefin derived from *Fasciola hepatica* has been identified in secretory products and shown to inhibit host cathepsin activity [13]. Similarly, recombinant *Fasciola gigantica* stefin-1 (rFgStefin-1) has been reported to exhibit anti-inflammatory effects by reducing the production of pro-inflammatory cytokines and mediators, including IL-1β, IL-6, IL-8, TNF-α, iNOS, and COX-2 [14]. However, despite its reported immunomodulatory activity, the potential role of rFgStefin-1 in leukemic cell biology and apoptosis-associated signaling has not yet been investigated.

In this study, we aimed to evaluate the effects of rFgStefin-1 on the human U937 leukemic cell line. Specifically, we examined its effects on cell viability and apoptosis to explore its potential as a biologically active molecule relevant to leukemia.

## Materials and methods

### Production of r*Fg*Stefin-1

The cDNA of stefin-1, Type I cystatin, of *Fasciola gigantica* (*Fg*Stefin-1) (accession no. FJ827152) was cloned into the pQE-30 expression vector (Qiagen, Hilden, Germany) and transformed into the M15 strain of *Escherichia coli* expression host as previously described [7,14]. Briefly, the positive clones of M15 *E. coli* containing pQE30/*Fg*Stefin-1 were used to ensure expression. The clones were ensured the correction of nucleotide sequence by colony PCR assay by using forward primer 5′-GGA TCC ATG ATG TGC GGC GGC TGC-3′ and reverse primer 5′-CTG CAG GAG TACCGA TCA TGA TC-3′. Then, the PCR-positive clones were selected for plasmid DNA extraction. The plasmid DNA was extracted using a QIAGEN Plasmid Mini Kit (Qiagen, Hilden, Germany), and the nucleotide sequencing was performed to confirm the correction (Solgent Co., Ltd., Daejeon, Korea). For r*Fg*Stefin-1 expression, the clones of M15 *E. coli* containing pQE30/*Fg*Stefin-1 were cultured until the OD_600_ reached 0.4–0.6 and induced with a final concentration of 1 mM isopropyl β-d-1-thiogalactopyranoside (IPTG) at 37 ◦C for 2 hours. The *E. coli* cells were harvested and ultrasonicated to facilitate protein extraction. The soluble r*Fg*Stefin-1 was separated by high-speed centrifugation at 10,000 rpm for 20 minutes at 4°C. Afterward, r*Fg*Stefin-1 was purified under native conditions using high-performance Ni Sepharose® (Cytiva, Uppsala, Sweden). The r*Fg*Stefin-1 were dialyzed against PBS, pH 7.4, and endotoxin was removed by using Triton X-114 (Sigma Aldrich, Darmstadt, Germany) for 10–15 rounds. Then, the endotoxin-free purified r*Fg*Stefin-1 concentration was measured by using a Pierce™ BCA protein assay kit (Thermo Fisher Scientific, Rockford, IL, USA). Finally, the expression of r*Fg*Stefin-1 was confirmed by western blot analysis using an anti-His tag antibody (Bio-Rad Laboratories, Inc., Hercules, CA, USA), and the protein was stored at −20°C until use.

### Cell culture

The human monocytic leukemic cell line (U937) was purchased from ATCC. This cell line is commonly used as a model of acute myeloid leukemia for studies of leukemic cell survival, differentiation, and apoptosis [15]. U937 cells were cultured in RPMI 1460 media (Hyclone, Cytiva, Marlborough, MA, USA) containing 10% fetal fetal bovine serum (FBS) and containing 100 U/mL antibiotic–antimycotic drug (Gibco, Life Technologies Corporation, Grand Island, NY, USA). The cell lines were cultured under 5% CO_2_ at 37°C and subcultured every 3–4 days.

### Peripheral mononuclear cells isolation

Consenting whole venous blood was collected from three healthy volunteers in BD vacutainer tubes with sodium heparin as an anticoagulant (BD Biosciences, Mississauga, ON, Canada), using protocols approved by The Research Ethics Review Committee for Research Involving Human Research Participants, Group I, Chulalongkorn University, with certificate of approval number COA. No. 199/68. The PBMCs were isolated by initially diluting whole blood in PBS (1:1 v/v), and then carefully layering the diluted blood suspension onto a Lymphoprep^TM^ density gradient medium (STEMCELL Technologies, Vancouver, BC, Canada). The PBMCs were centrifuged at 800 *× g* for 30 minutes at room temperature. After the centrifugation step, PBMCs were removed, and the upper plasma layer was discarded without disturbing the plasma: Lymphoprep^TM^ interface. PBMCs were transferred into a new tube. Subsequently, the cells were centrifuged and washed twice with PBS and once with RPMI-1640 medium supplemented with 10% FBS. The PBMCs were resuspended in medium and incubated in a flask at 37°C with 5% CO_2_ for 1 hour prior to investigating the cytotoxicity assay.

### Cell cytotoxicity assay

The cytotoxic effect of rFgStefin-1 on U937 leukemic cell lines and normal PBMCs was investigated using the XTT assay (Abcam, Cambridge, EN, UK) as previously described [16]. Briefly, U937 and PBMC cells were seeded at 1 × 10^4^ cells/well in a 96-well plate and treated with varying concentrations of r*Fg*Stefin-1 (1.09375, 2.1875, 4.375, 8.75, 17.5, 35, and 70 μM) compared with CA-074Me, a cathepsin B inhibitor. The cells were cultured under 5% CO_2_ at 37°C for 24 and 48 hours. After treatments, 1 mg/μl of XTT working solution containing 7.5 μg/ml PMS (Sigma-Aldrich, Germany) was added to each well, and the cultures were incubated at 37°C for 4 hours. The optical density of the formazan product (450 nm) and the reference (690 nm) was measured for each well using a Multiskan Spectrophotometer (Thermo Scientific, Rockford, IL, USA). The percentage of cell viability was calculated from the following formula: (Mean Absorbance of Treated Cells / Mean Absorbance of Control Cells) x 100%. The data are presented as the mean percentage of cell viability ± standard deviation (SD) from three independent experiments performed in triplicate. The half-maximal inhibitory concentration (IC_50_) was determined using GraphPad Prism (GraphPad Software, San Diego, CA, USA).

### Analysis of mitochondrial membrane potential alteration

The alteration in mitochondrial membrane potential was measured using JC-1 dye as previously described [17]. Briefly, U937 cells at 1.5 × 10^5^ cells/well were cultured in a 24-well plate and treated with r*Fg*Stefin-1 at concentrations of 5.5 μM (0.5X IC_50_), 11 μM (1X IC_50_), and 22 μM (2X IC_50_). After 24 hours of treatment, the cells were collected, and the medium was removed by centrifugation at 1,500 rpm for 5 minutes at 4°C. After that, pallet cells were resuspended in RPMI-1640 medium supplemented with 10% FBS and stained with 2.5 μg/ml of JC-1 dye for 20 min at 37°C in a humidified atmosphere containing 5% CO_2_. The stained U937 cells were washed twice in cold PBS and resuspended in 300 μL ice-cold JC-1 staining buffer. For assessment of r*Fg*Stefin-1-treated cells for JC-1 staining, a total of 10,000 events were measured for each sample using the DxFLEX flow cytometry system (Beckman Coulter, Brea, CA, USA) with 488 nm as a laser excitation wavelength. To quantify the population of U937 cells, the emission wavelengths of JC-1 monomers (535 nm) and JC-1 aggregates (595 nm) were used to detect green and red fluorescence, respectively. The percentages of cells exhibiting polarized or depolarized mitochondria were assessed by histogram analysis of the red-to-green fluorescence intensity ratio. The numbers represent U937 cells expressing only green monomer fluorescence. The data represented the means of three independent experiments performed in triplicate.

### Apoptosis determination

U937 cell apoptosis was performed according to the manufacturer’s instructions of Annexin V-FITC/Propidium iodide (PI) apoptosis kit (Invitrogen, USA), with slight modifications. Briefly, U937 cells at 1.5 × 10^5^ cells/well were cultured in a 24-well plate in the presence of r*Fg*Stefin-1 at concentrations of 5.5 μM, 11 μM, and 22 μM for 24 hours. The cells were harvested by centrifuging at 1,500 rpm for 5 minutes. The cell pellet was resuspended in 100 μL 1X annexin binding buffer and stained with 2.5 μL Annexin V-FITC, followed by 1 μL PI. Cells were mixed and incubated for 15 minutes in dark conditions. Then, 400 μL of 1X annexin-binding buffer was added and kept on ice until analysis on the DxFLEX flow cytometry system (Beckman Coulter, Brea, CA, USA). At least 10,000 events were collected for each sample. The distribution of apoptotic stages among treated cells was expressed as the percentage of cells in early and late apoptotic stages.

### mRNA expression analysis

After treatment of the U937 cells at 2 × 10^6^ cells with r*Fg*Stefin-1 at 5.5 μM, 11 μM, and 22 μM for 48 hours, total RNA was extracted using TRIzol reagent (Invitrogen, Carlsbad, CA, USA) according to the manufacturer’s instructions. Then, the mRNA was reverse transcribed to first-strand cDNA using the SuperScript™ III First-Strand Synthesis System (Thermo Scientific, USA). The mRNA expression levels of BAX, BCL2, CASP3 (Caspase-3), PARP1, PI3K, AKT1, and GAPDH (a housekeeping control) were investigated. qRT-PCR was performed to quantify mRNA levels by using iTaq Universal SYBR Green Supermix (Bio-Rad Laboratories) on a StepOne™ Real-Time PCR System (Applied Biosystems, Foster City, CA, USA). Relative mRNA quantification was analyzed using 2^−∆∆CT^, with GAPDH as the housekeeping gene. The values are reported as the fold changes relative to the control. The primer sequences used for the real-time PCR are listed in **Table 1**.

**Table 1:** The primer sequences used for the reverse transcription quantitative real-time polymerase chain reaction (qRT-PCR).

Gene		Primer Sequence	Product size (bp)
*BCL2*	FW:	ATCGCCCTGTGGATGACTGAGT	127
	RV:	GCCAGGAGAAATCAAACAGAGGC	
*BAX*	FW:	TGGCAGCTGACATGTTTTCTGAC	195
	RV:	TCACCCAACCACCCTGGTCTT	
*CASP3*	FW:	GGAAGCGAATCAATGGACTC	146
	RV:	GCATCGACATCTGTACCAGA	
*PARP1*	FW:	AAGCCAGTTCAGGACCTCAT	119
	RV:	ATCTGCCTTTTGCTCAGCTT	
*PI3K*	FW:	GAATTGGGAGAACCCAGACA	219
	RV:	GAATTTCGCACCACCTCAAT	
*AKT1*	FW:	CATCACACCACCTGACCAAG	201
	RV:	CTCAAATGCACCCGAGAAAT	
*GAPDH*	FW:	GAGTCAACGGATTTGGTCGT	214
	RV:	TGGAAGATGGTGATGGGATT	

### Western blot analysis

U937 cells at 2 × 10^6^ cells were treated with the r*Fg*Stefin-1 at 5.5 μM, 11 μM, and 22 μM for 48 hours. Then the cellular proteins were extracted. The cells were lysed by RIPA cell lysis buffer (Cell Signaling Technology®, USA) containing protease inhibitors (Merck Millipore Calbiochem^TM^ Protease Inhibitor Cocktail Set III, EDTA-Free, Germany). The protein concentration was measured using Pierce^TM^ BCA Protein Assay Kit (Thermo Fisher Scientific Inc., Rockford, IL, USA). Each sample, equal to 20 μg, was subjected to 12.5% sodium dodecyl sulfate-polyacrylamide gel electrophoresis (SDS-PAGE) and transferred onto 0.45 µM nitrocellulose membranes (Cytiva, Birmingham, UK). The non-specific bindings were blocked using 5% BSA in tris-buffered saline (TBS), pH 7.5, for 1 hour at room temperature and incubated with 1:1,000 primary antibodies diluted in 1% BSA in TBS with 0.1% (v/v) Tween®-20 (TBST), which involved anti-PI3K, anti-Akt1, anti-p-Akt, anti-cleaved PARP, anti-cleaved caspase-3 (Cell Signaling Technolog®, USA), anti-Bcl-2, anti-Bax and anti-GAPDH (Abcam, USA) overnight at 4°C. The membranes were washed three times in TBST and subsequently incubated for 1 hour at room temperature with a 1:15,000 Goat anti-Rabbit IgG (H + L) Secondary Antibody, AP (Abcam, USA). Following that, Proteins of interest were visualized using 1-Step™ NBT/BCIP Substrate Solution (Thermo Scientific, Rockford, IL). The protein band intensity was analyzed using ImageJ, and GAPDH was normalized and used as an internal control.

### Statistical analyses

The data represent mean ± SD from three independent experiments performed in triplicate. One-way analysis of variance (ANOVA) was performed to assess differences among quantitative variables, followed by Dunnett’s post hoc multiple comparison test to identify significant differences between groups. Statistical tests and graphical representations of the data were generated using xGraphPad Prism software version 9.3.1. (GraphPad Software, San Diego, CA, USA). A *p*-value less than 0.05 was considered statistically significant.

## Results

### The expression of r*Fg*Stefin-1

The r*Fg*Stefin-1 was successfully constructed in the pQE-30 expression vector and expressed in the expression host, *Escherichia coli* M15 strain, as previously described [7,14]. The r*Fg*Stefin-1 was produced in an *E. coli* expression system using IPTG induction for 2 hours (S1A Fig). High yields of purified r*Fg*Stefin-1 protein were obtained using Ni Sepharose affinity chromatography, particularly in the E1–E2 fractions (S1B Fig). SDS–PAGE analysis of the purified r*Fg*Stefin-1 revealed two major bands at 11 kDa and 22 kDa, corresponding to the monomeric and dimeric forms, respectively. Additionally, the purified, dialyzed, and endotoxin-removed r*Fg*Stefin-1 showed the same pattern as in S1C Fig in the SDS-PAGE analysis (S1C Fig).

### The cytotoxic effect of r*Fg*stefin-1

**Fig 1:**
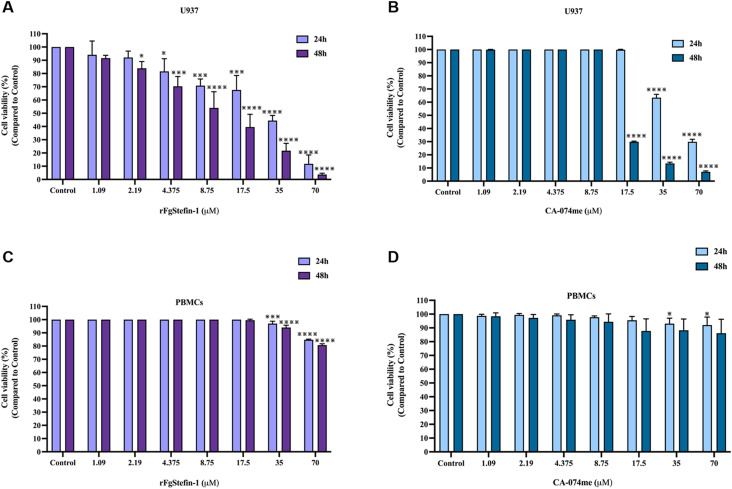
Cytotoxic effects of rFgStefin-1 on U937 cells. Cell viability was assessed using the XTT assay. U937 cells were treated with different concentrations of rFgStefin-1 (A) or CA-074Me (B) for 24 and 48 h. Normal PBMCs were treated under the same conditions with rFgStefin-1 (C) or CA-074Me (D). Data are presented as mean ± SD from three independent experiments (n = 3). Statistical significance was determined using one-way ANOVA followed by Dunnett’s post hoc test (*p < 0.05, **p < 0.01, ***p < 0.001, ****p < 0.0001).

**Fig 2:**
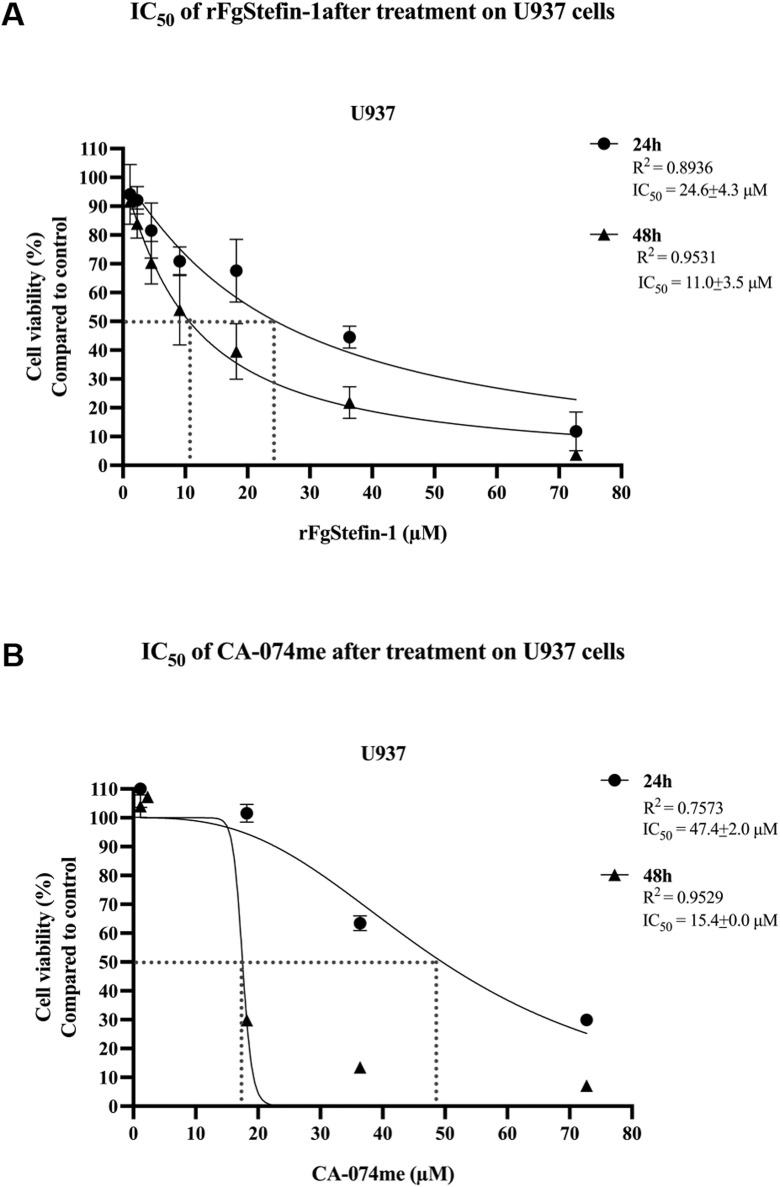
Dose–response analysis of rFgStefin-1 in U937 cells. Nonlinear regression curves were generated to determine the IC₅₀ values of rFgStefin-1 (A) and CA-074Me (B) in U937 cells following 24 and 48 h of treatment.

Previous studies have reported that r*Fg*Stefin-1 acts as a cathepsin inhibitor, specifically inhibiting cathepsin B, S, and L [7,18]. Here, we first examined the effect of r*Fg*Stefin-1 on leukemic cell viability. U937 monocytic leukemic cells were treated with r*Fg*Stefin-1, vehicle, or CA-074Me for 24 and 48 hours, and the cytotoxic effect was measured by XTT assay. After 24 hours of treatment, U937 cells treated with r*Fg*Stefin-1 at 4.375 μM showed a significant initial decrease in cell viability to 81.5% (*p* < 0.05) compared to the control (Fig 1A). In contrast, treatment with CA-074Me at the same concentration did not reduce cell viability (Fig 1B). Interestingly, after 48 hours of treatment, U937 cells treated with r*Fg*Stefin-1 at 8.75 μM showed a significant decrease in cell viability to 54.1% (*p* < 0.0001), compared to the control. In contrast, treatment with the same concentration of CA-074Me did not affect U937 cell viability. The non-linear regression dose-response graphs used for IC_50_ analysis of r*Fg*Stefin-1 and CA-074Me against U937 cell lines at 24 and 48 hours of treatment are also demonstrated in Fig 2A and 2B. After 24 hours of treatment, the IC_50_ of r*Fg*Stefin-1 and CA-074Me against U937 cells were 24.6 μM and 47.4 μM, respectively. After 48 hours of treatment, the IC_50_ of r*Fg*Stefin-1 and CA-074Me against U937 cells were 11.0 μM and 15.4 μM, respectively. Moreover, the cell viability of r*Fg*Stefin-1- and CA-074Me-treated peripheral blood mononuclear cells (PBMCs) was investigated, revealing no toxicity, especially at the IC_50_ concentration, as shown in Figs 1C and 1D. These data suggest that r*Fg*Stefin-1 significantly decreased the cell viability of monocytic U937 leukemic cells. In contrast, these compounds showed no cytotoxicity on PBMCs.

### The Mitochondrial membrane potential alteration

**Fig 3:**
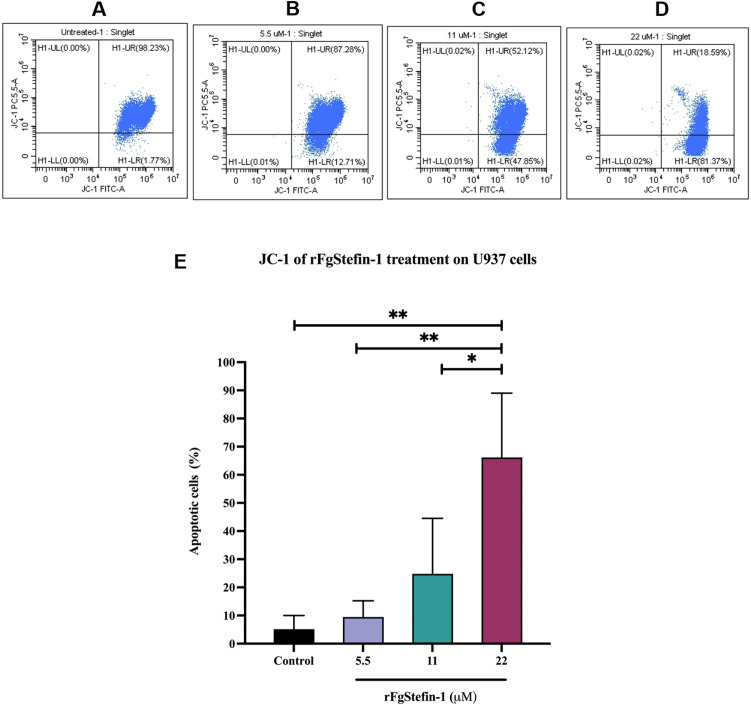
Effect of rFgStefin-1 on mitochondrial membrane potential in U937 cells. Representative JC-1 flow cytometry dot plots of U937 cells treated with rFgStefin-1 for 24 h: (A) control, (B) 5.5 µM, (C) 11 µM, and (D) 22 µM. (E) Quantification of cells with depolarized mitochondria based on JC-1 staining. Data are presented as mean ± SD (n = 3). Statistical significance was determined using one-way ANOVA followed by Dunnett’s post hoc test (*p < 0.05, **p < 0.01).

We further assessed the effect of r*Fg*Stefin-1 on Mitochondrial membrane potential (MMP) alterations, as a reduction in MMP may also be linked to apoptosis [19]. The alteration in mitochondrial membrane potential occurred during the early stage of caspase-induced apoptosis. In healthy cells, the cationic carbocyanine JC-1 dye predominantly exists as an aggregated form in the mitochondria, which appears as red fluorescence. In apoptotic cells, JC-1 dye predominantly exists as a monomeric form, which emits green fluorescence. As shown in Fig 3A-3D, dot plots analyzed by flow cytometry for JC-1-stained U937 cells after 24 hours of treatment with r*Fg*Stefin-1 revealed an elevated presence of green-fluorescing monomers in the lower quadrant, indicating increased apoptotic cells. The percentage of apoptotic U937 cells after treatment with rFgStefin-1 is also shown in Fig 3E. In the control group, approximately 5.2% of U937 cells were apoptotic. After treatment with 5.5, 11, and 22 μM of r*Fg*Stefin-1, the percentage of apoptotic U937 cells increased in a dose-dependent manner, reaching 9.5%, 24.8%, and 66.2%, respectively. At 22 μM rFgStefin-1, the percentage of apoptotic cells was significantly higher than in the control (*p* < 0.01). These results demonstrated that the r*Fg*Stefin-1 exhibited potent anti-leukemic activity by disrupting mitochondrial membrane potential, thereby inducing apoptosis.

### Apoptosis determination

**Fig 4:**
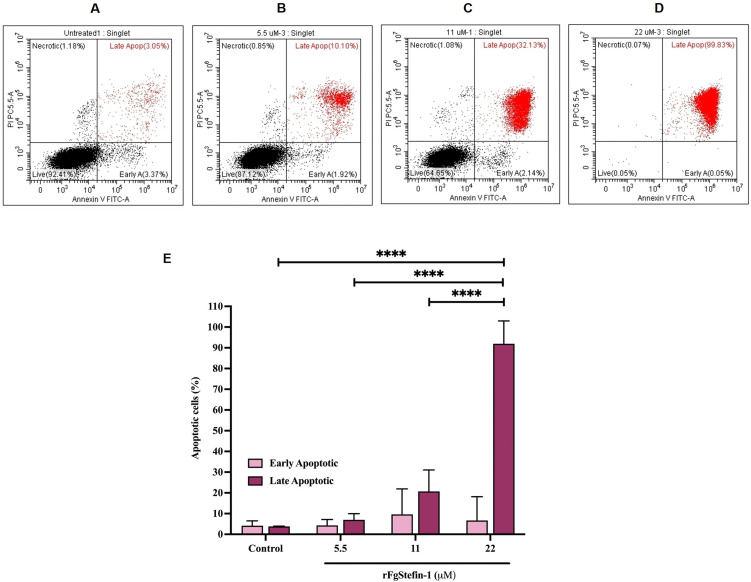
Effect of rFgStefin-1 on apoptosis in U937 cells. U937 cells were treated with increasing concentrations of rFgStefin-1 for 24 h. **(A–D)** Representative Annexin V-FITC/PI flow cytometry plots of control, 5.5, 11, and 22 µM-treated cells, respectively. **(E)** Quantification of early and late apoptotic cell populations. Data are presented as mean ± SD from three independent experiments (n = 3). Statistical significance was determined using one-way ANOVA followed by Dunnett’s post hoc test (****p < 0.0001).

Since r*Fg*Stefin-1 modulates mitochondrial membrane potential alteration, we further confirmed whether it could induce apoptosis in U937 cells. We determined the apoptotic cell death induced by recombinant protein treatment in monocytic leukemic cells using annexin V/Propidium Iodide (PI) double staining by flow cytometry. As shown in Fig 4A-4D, r*Fg*Stefin-1 significantly induced U937 cell apoptosis after 24 hours of treatment. In the control group, the untreated cell population contained a low number of cells in both the early and late stages of apoptosis, as indicated by the scatter plot distribution in the right quadrant. As shown in **Fig 4E**, the percentage of late-apoptotic U937 cells increased in a dose-dependent manner, reaching approximately 7.0%, 20.7%, and 92.0% at concentrations of 5.5, 11, and 22 μM, respectively, compared with 3.8% occured in the control group. In particular, at 22 μM r*Fg*Stefin-1, the percentage of late apoptotic cells was significantly higher than in the other groups (*p* < 0.0001). These results indicated that r*Fg*Stefin-1 promoted apoptosis in leukemic cells.

### The mRNA expression by reverse transcription quantitative real-time polymerase chain reaction (qRT-PCR) analysis

**Fig 5:**
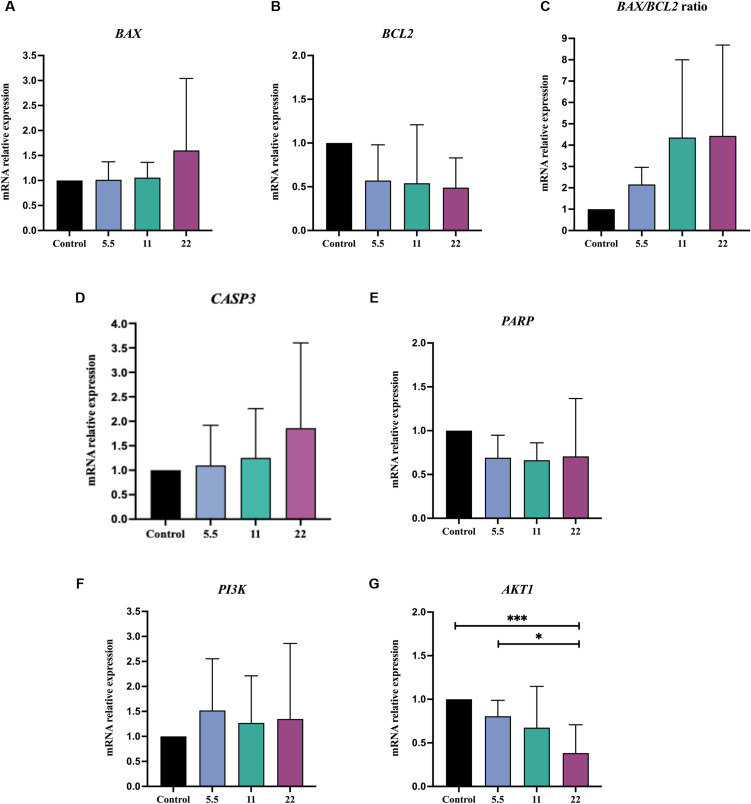
Effect of rFgStefin-1 on apoptosis- and survival-related gene expression in U937 cells. U937 cells were treated with increasing concentrations of rFgStefin-1 for 48 h. Relative mRNA expression levels of **(A)** BAX, **(B)** BCL2, **(C)** BAX/BCL2 ratio, **(D)** CASP3, **(E)** PARP1, **(F)** PI3K, and **(G)** AKT1 were determined by qRT-PCR. Data are presented as mean ± SD from three independent experiments (n = 3). Statistical significance was determined using one-way ANOVA followed by Dunnett’s post hoc test (*p < 0.05, **p < 0.01, ***p < 0.001).

Because the recombinant protein reduced the mitochondrial membrane potential and induced apoptosis in U937 cells, it may serve as a potential therapeutic agent against leukemia. In order to examine the r*Fg*Stefin-1-induced alteration of proapoptotic-related molecules, including *BAX*, *CASP3*, and *PARP1* and anti-apoptotic effectors, including *BCL2*, *PI3K*, and *AKT1,* we investigated the mRNA expression of the mentioned molecules by qRT-PCR. In a dose-dependent manner, the treated U937 cells strongly upregulated these proapoptotic-related molecules and downregulated anti-apoptotic effectors, as shown in **Fig 5**. The 22 μM r*Fg*Stefin-1 altered the levels of augmentation molecules in *BAX* (~1.6-fold higher than controls) and *BCL2* (~0.5-fold lower than controls). Analysis of the *BAX/BCL2* ratio, a recognized prognostic indicator in leukemia, showed that an elevated ratio is associated with improved therapeutic response, consistent with previous reports linking this ratio to enhanced apoptosis of leukemic cells [20]. Notably, we found that the *BAX/BCL2* ratio was elevated in a dose-dependent manner, particularly at 11 and 22 μM rFgStefin-1, which were approximately 3.6- and 4.4-fold higher than the control, respectively (**Fig 5C**). At 22 μM r*Fg*Stefin-1, leukemic cell apoptosis was induced, which may involve its effects on *CASP3* mRNA overexpression (~1.9-fold higher than controls), as shown in **Fig 5D**. Interestingly, a 22 μM concentration of rFgStefin-1 likely induced apoptosis through reduced AKT1 mRNA expression (~0.4-fold lower than controls), with statistical significance (*p* < 0.001), as shown in **Fig 5G***.*

### Western blot analysis

**Fig 6:**
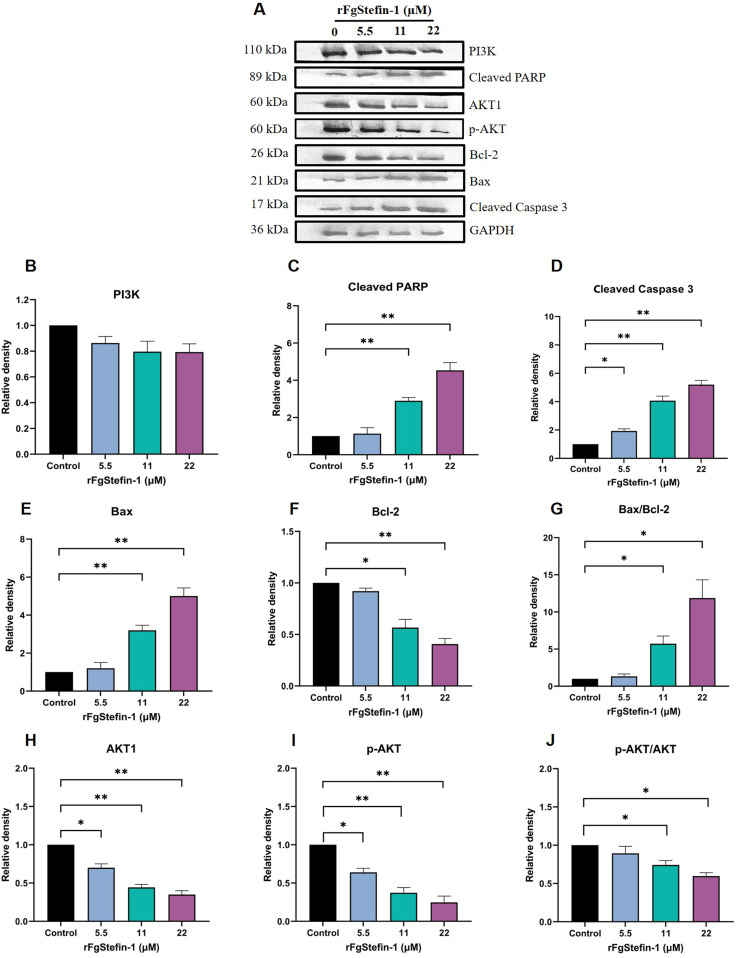
Effect of rFgStefin-1 on apoptosis- and survival-related protein expression in U937 cells. U937 cells were treated with increasing concentrations of rFgStefin-1 for 48 h. **(A)** Representative Western blot images showing PI3K, cleaved PARP, AKT1, p-AKT, Bcl-2, Bax, and cleaved caspase-3, with GAPDH used as a loading control. **(B–J)** Densitometric analysis of PI3K, cleaved PARP, cleaved caspase-3, Bax, Bcl-2, Bax/Bcl-2 ratio, AKT1, p-AKT, and p-AKT/total AKT ratio, respectively. Protein expression levels were quantified using ImageJ and normalized to GAPDH. Data are presented as mean ± SD from three independent experiments (n = 3). Statistical significance was determined using one-way ANOVA followed by Dunnett’s post hoc test (*p < 0.05, **p < 0.01).

To assess whether the mRNA changes translated into protein expression, Western blot analysis was performed. The same targets analyzed by qRT-PCR were included, covering pro-apoptotic markers (BAX, cleaved CASP3, and cleaved PARP1) and anti-apoptotic/survival-related proteins (BCL2, PI3K, and AKT1) (**Fig 6A**). This allowed comparison between gene expression and protein levels after rFgStefin-1 treatment. After treatment of the U937 cells with the r*Fg*Stefin-1 at 5.5 μM (0.5x IC_50_), 11 μM (1x IC_50_), and 22 μM (2x IC_50_) for 48 hours, PI3K expression showed a decreasing trend across all concentrations; however, the difference was not statistically significant (**Fig 6B**). In contrast, cleaved CASP3 levels were significantly increased at all tested doses (**Fig 6D**). Even PARP1 mRNA levels did not show a significant change in the qRT-PCR analysis, whereas at the protein level, cleaved PARP was significantly elevated at 11 and 22 µM but not at 5.5 µM (**Fig 6C**). AKT1 expression was significantly reduced at all concentrations (**Fig 6H**). For apoptosis-related proteins, BAX expression was significantly increased at 11 and 22 µM (**Fig 6E**), while BCL2 expression was significantly decreased at the same concentrations (**Fig 6F**). Consistently, the BAX/BCL2 ratio was significantly increased at 11 and 22 µM (**Fig 6G**).

Treatment with rFgStefin-1 reduced both total AKT (**Fig 6H**) and phosphorylated AKT levels (**Fig 6I**) in U937 cells. The results further showed a decrease in the p-AKT/total AKT ratio (**Fig 6J**), indicating reduced AKT activation following rFgStefin-1 treatment. These findings suggest that rFgStefin-1-induced apoptosis may be associated, at least in part, with suppression of AKT-mediated survival signaling

## Discussion

Cysteine cathepsin proteases have been reported to be overexpressed in various diseases, including cancer, especially in solid tumors as well as leukemia. Overexpression of cathepsins B, S, and L in leukemia has been reported to be associated with an unfavorable prognosis in patients [21]. The biological activity of cysteine cathepsin proteases is normally regulated by endogenous cysteine protease inhibitors, the cystatins, which comprise several families. Stefin-1 is a member of the type-I cystatin family, which is identified among several organisms, including protozoa, helminths, animals, and humans. Extracellular cystatins are implicated in host defense mechanisms by neutralizing circulating cysteine proteases originating from both endogenous and pathogen-derived sources [22].

In *F. gigantica*, stefin-1 (*Fg*Stefin-1) is a prominent secreted antigen existing in the excretory/secretory products of the adult parasite*.* A prior study demonstrated the conservation of *Fg*Stefin-1 within the same protein family, and the successful production of recombinant stefin-1 derived from *F. gigantica* (r*Fg*Stefin-1) prompted them to test its prominent activities. The *Fg*Stefin-1 protein has been characterized and shown to inhibit the activity of mammalian cathepsins B, S, and L [7,18]. Later, Chantree et al. showed that r*Fg*Stefin-1 exerts anti-inflammatory effects via the NF-κB signaling pathway in the macrophages [14]. However, the anti-cancer activities of *Fg*Stefin-1 have never been reported. Hence, this study is the first to demonstrate that r*Fg*Stefin-1 inhibits leukemia cell proliferation by inducing mitochondrial membrane dysfunction, thereby triggering caspase-mediated apoptosis in human U937 leukemia cells.

In this study, we demonstrated that r*Fg*Stefin-1, at concentrations ranging from 4.375–70 μM, significantly reduced the viability of U937 cells (**Fig 1A**). Furthermore, no cytotoxic effects were observed in normal PBMCs treated with r*Fg*Stefin-1 (**Fig 1C and 1D**). Our findings indicated that r*Fg*Stefin-1 is selective for cancer cells, without affecting normal cells, particularly within the concentration range of our present study. Moreover, it is correlated with a previous study, which indicated that r*Fg*Stefin-1 exhibits anti-inflammatory activity at concentrations between 0.45 and 1.82 μM (5–20 µg/mL) without affecting macrophages, RAW 264.7 and THP-1 cell viability. Otherwise, the observed anti-inflammatory effects were linked to the downregulation of pNF-κB and reduced secretion of COX-2, IL-1β, IL-6, and TNF-α [14]. The previous report has prompted our interest in the anti-leukemic activity of r*Fg*Stefin-1, given its related intracellular functions observed in macrophages. Several natural compounds have been identified as having both anti-inflammatory and anti-cancer activities. Intensive research has focused on the potential of anti-inflammatory agents, especially COX-2 inhibitors, as adjuvants in chemotherapy [23]. Among them, Celecoxib has been shown to have the most significant synergistic effects in enhancing chemotherapy. Celecoxib exerts its effects by inhibiting COX-2 expression and reducing levels of IL-8, TNF-α, NF-κB, and apoptosis-related proteins [24,25]. So, it is more likely that r*Fg*Stefin-1 could have effects similar to those of the anti-inflammatory agents, but the involved pathways will be further explored.

Interestingly, our cytotoxic findings showed that r*Fg*Stefin-1 can inhibit cancer growth more effectively than CA-074Me, a cathepsin B inhibitor, at comparable concentrations ranging from 1.09 to 70 μM (**Fig 1B**). Leukemia cells overexpress cathepsins B and L, and previous studies have shown that CA-074Me can inhibit both kinds of cysteine proteases. However, its effectiveness against cathepsin L is diminished under reducing intracellular conditions, limiting its specificity *in vivo* [26]. Surprisingly, in this present study, the cytotoxic activity of CA-074Me is lower than that of r*Fg*Stefin-1, potentially due to the effects of neutral pH on cathepsin B inhibition [27]. However, this finding indicated that the r*Fg*Stefin-1 may have a better cytotoxic effect on U937 cells than the cathepsin B inhibitor. It is indicated that r*Fg*Stefin-1 has potential as an anti-leukemic agent. This finding leads us to investigate the deep mechanisms occurring intracellularly.

Not only were cytotoxicity effects observed, as reported, but we also observed morphological changes in the treated cells. We observed apoptotic features in the r*Fg*Stefin-1-treated U937 cells. Apoptosis is a common form of cell death that progresses through early and late stages, with the intactness or permeability of the cell membrane serving as a key distinguishing feature. We subsequently explore the mechanisms underlying r*Fg*Stefin-1-induced cell death in U937 cells. To investigate this, we first evaluated the impact of r*Fg*Stefin-1 on changes in mitochondrial membrane potential (**Fig 3**). In recent years, numerous studies have focused on mitochondria as a key target for inducing chemotherapy-mediated apoptosis in acute myeloid leukemia [28]. Several studies have demonstrated that the regulation of mitochondria-related apoptosis is intricately linked to Bcl-2 family proteins. In the absence of apoptotic stimuli, Bax is localized in the cytosol and the outer mitochondrial membrane. Upon the induction of death signals, Bax accumulates at the outer mitochondrial membrane, where it interacts with both the voltage-dependent anion channel (VDAC) and the adenine nucleotide carrier. This interaction leads to the formation of the permeability transition pore, resulting in decreased mitochondrial membrane potential, mitochondrial swelling, outer membrane rupture, and release of cytochrome c [29]. In the present study, mitochondrial membrane depolarization together with altered Bax/Bcl-2-associated signaling collectively supported the involvement of mitochondrial-associated apoptotic mechanisms in rFgStefin-1-treated U937 cells. However, caspase-9 activation was not evaluated in the current study. Therefore, although the findings are consistent with intrinsic apoptotic signaling, additional studies examining upstream mitochondrial apoptotic mediators, including caspase-9 activation, would further strengthen mechanistic interpretation.

Apoptosis was confirmed through Annexin V/Propidium Iodide (PI) double staining followed by flow cytometric analysis (**Fig 4**). The proportion of late-apoptotic U937 cells increased in a dose-dependent manner, with a significant elevation observed at 22 μM r*Fg*Stefin-1 compared with other treatment groups. These findings support the conclusion that r*Fg*Stefin-1 induces apoptosis in leukemic cells. The apparent discrepancy between the observed apoptotic cell population and the calculated IC50 value may be related to differences in experimental endpoints and treatment durations. In the present study, Annexin V-FITC/PI and JC-1 assays were performed after 24 h of treatment, whereas XTT-based viability analysis and IC50 determination were conducted after 48 h. Furthermore, the XTT assay primarily reflects cellular metabolic activity, whereas Annexin V-based flow cytometry directly evaluates apoptotic cell death. Therefore, differences in assay sensitivity and biological endpoints may contribute to the variation observed between apoptotic cell percentages and metabolic viability measurements. Our findings are consistent with a previous study using recombinant human cystatin. The recombinant stefin-1, derived from human cystatin A, was synthesized and transfected into H2170 lung cancer cell lines. The human recombinant stefin-1 was found to reduce cathepsin B activity, suppress colony formation and migration, and enhance gemcitabine-induced apoptosis [8].

Recent research has demonstrated that Bcl-2 functions as an anti-apoptotic protein, contributing significantly to the resistance of cancer cells to chemotherapy [30]. The Bax/Bcl-2 ratio has been established as a critical indicator of apoptosis, reflecting the equilibrium between pro-apoptotic and anti-apoptotic signals. A high Bax/Bcl-2 ratio is associated with an increased apoptotic cell population, whereas a low ratio correlates with enhanced cell survival and resistance to apoptosis [31]. In this study, we showed that r*Fg*Stefin-1-induced apoptosis is associated with upregulation of Bax and downregulation of Bcl-2. Our findings further suggest that the increased Bax/Bcl-2 ratio is a key determinant of apoptosis, correlates with mitochondrial dysfunction, and triggers apoptosis in U937 cells. Moreover, we have demonstrated that r*Fg*Stefin-1 induces apoptosis by upregulating caspase-3. Furthermore, r*Fg*Stefin-1 increased the level of cleaved PARP. PARP is normally involved in DNA repair, whereas its cleaved form is widely recognized as a marker of apoptosis [32]. Therefore, these data indicated that r*Fg*Stefin-1-induced apoptosis was caused by caspase-3-dependent cell death. Moreover, our results demonstrated that rFgStefin-1 reduced both total AKT and phosphorylated AKT expression levels in U937 cells, accompanied by a decreased p-AKT/AKT ratio **(Figs 5 and 6)**. Since AKT signaling plays an important role in regulating leukemic cell survival and apoptosis resistance [33], reduced AKT activation may contribute to the apoptotic effects observed following rFgStefin-1 treatment. It is consistent with a previous study that demonstrated caspase-3-mediated cleavage of Akt1 [34]. Akt1 plays an important role in regulating NF-κB, promoting cell survival and proliferation [35]. Although PI3K showed a decreasing trend without statistical significance, the consistent downregulation of Akt1 may still reflect an overall attenuation of pro-survival signaling. Given the central role of Akt in promoting cell survival and inhibiting apoptosis, its reduction may contribute to the activation of apoptotic processes observed in this study.

Although the present findings demonstrated apoptosis induction and modulation of AKT-associated survival signaling, the direct molecular target responsible for initiating these effects remains unidentified. Additional studies using pull-down assays, affinity-based protein interaction analysis, co-immunoprecipitation, and functional validation approaches would help clarify the upstream molecular mechanisms underlying rFgStefin-1-induced apoptosis.

Differences between mRNA expression and protein-level findings observed in this study may reflect the complex multi-layered regulation of apoptosis- and survival-related signaling pathways [36]. Transcript expression determined by qRT-PCR does not necessarily correlate directly with protein abundance or activation status, as post-transcriptional regulation, translational efficiency, protein turnover, and proteolytic processing can substantially influence protein expression patterns [37]. For example, increased cleaved caspase-3 and cleaved PARP levels represent activation of apoptotic execution pathways rather than simple transcriptional upregulation [38,39]. Similarly, the absence of a marked change in PARP1 mRNA despite increased cleaved PARP protein levels may reflect apoptosis-associated proteolytic cleavage [40]. These findings suggest that rFgStefin-1-induced apoptosis involves both transcriptional and post-translational regulatory mechanisms.

Although the present study demonstrated selective apoptosis-inducing effects of rFgStefin-1 in U937 cells, leukemia is biologically heterogeneous with substantial differences in lineage, differentiation status, molecular background, and therapeutic responsiveness [41]. Therefore, further validation using additional leukemia models, including AML and therapy-resistant leukemic cell lines, would be important to determine the broader anti-leukemic applicability of rFgStefin-1.

In addition, the present study mainly focused on apoptosis induction. However, leukemic progression and therapeutic resistance are also associated with other biological characteristics, including leukemic stemness, differentiation status, migration, adhesion-mediated survival, and interactions with the bone marrow niche [42,43]. Future studies investigating these leukemia-associated phenotypes would further expand understanding of the anti-leukemic potential of rFgStefin-1.

**Fig 7:**
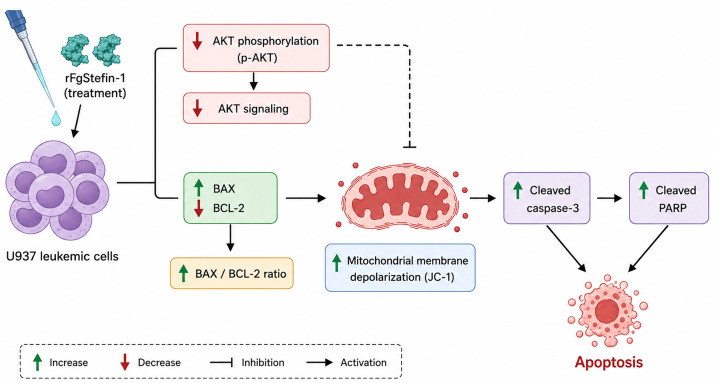
Proposed mechanism of rFgStefin-1-induced apoptosis in U937 leukemic cells based on the present findings. Treatment with rFgStefin-1 was associated with reduced AKT phosphorylation and decreased AKT-associated survival signaling, accompanied by increased BAX expression, decreased BCL-2 expression, and an elevated BAX/BCL-2 ratio. These molecular alterations were associated with mitochondrial membrane depolarization, as demonstrated by JC-1 staining, followed by increased cleaved caspase-3 and cleaved PARP expression, ultimately leading to apoptosis in U937 cells. The schematic summarizes the proposed mitochondrial-associated apoptotic signaling pathway based on the experimental results obtained in this study.

Our data provide new evidence that the helminth-derived cystatin rFgStefin-1 promotes apoptosis in U937 leukemic cells through mechanisms associated with mitochondrial apoptotic signaling, including altered Bax/Bcl-2-associated regulation, mitochondrial membrane depolarization, activation of caspase-3, PARP cleavage, and suppression of AKT-mediated survival signaling (Fig 7). Nevertheless, additional mechanistic studies, validation in multiple leukemia models, and *in vivo* investigations will be necessary before considering potential translational applications of rFgStefin-1.

## Conclusion

This study is the first to demonstrate that recombinant Stefin-1 derived from *Fasciola gigantica* (rFgStefin-1), a helminth-derived cysteine protease inhibitor, reduces leukemic cell viability and promotes apoptosis in U937 cells. The apoptotic effects of rFgStefin-1 were associated with mitochondrial membrane depolarization, increased cleaved caspase-3 and cleaved PARP expression, altered Bax/Bcl-2-associated signaling, and reduced AKT-associated survival signaling. These findings provide preliminary evidence supporting the potential biological activity of rFgStefin-1 in leukemia-related contexts. Nevertheless, further mechanistic studies, validation in additional leukemia models, and in vivo investigations are required before considering potential translational applications.

## Supporting information

S1 Fig SDS-PAGE analysis of r*Fg*Stefin-1 expression in an *E. coli* expression system. (A) Four clones of *E. coli* M15 containing pQE-30/*Fg*Stefin-1 were induced with 1 mM IPTG for 2 hours (2H), compared with non-induced (NI). (B) Purification of r*Fg*Stefin-1 by high-performance Ni Sepharose® (Cytiva, Uppsala, Sweden). using native conditions. (C) SDS-PAGE of purified r*Fg*Stefin-1 before and after endotoxin removal from the different batches. M: Whole Blue Range Prestained Protein Ladder (Vivantis, Malaysia); SP: Soluble protein fraction before purifying; FT: flow through; W_1_: wash fraction-1; W_2_: wash fraction-2; E_1_-E_5_: elution fraction 1–5.
(TIF)

S2 Fig Original uncropped western blot images underlying the western blot results presented in this study. (PDF)
