# Supplementary figures and images for: Helminth-derived stefin-1 selectively reduces leukemic cell viability and promotes apoptosis in U937 cells
Source: PLoS One. 2026 Jul 27;21(7):e0353364. doi: 10.1371/journal.pone.0353364 (PMC13405116; doi:10.1371/journal.pone.0353364)

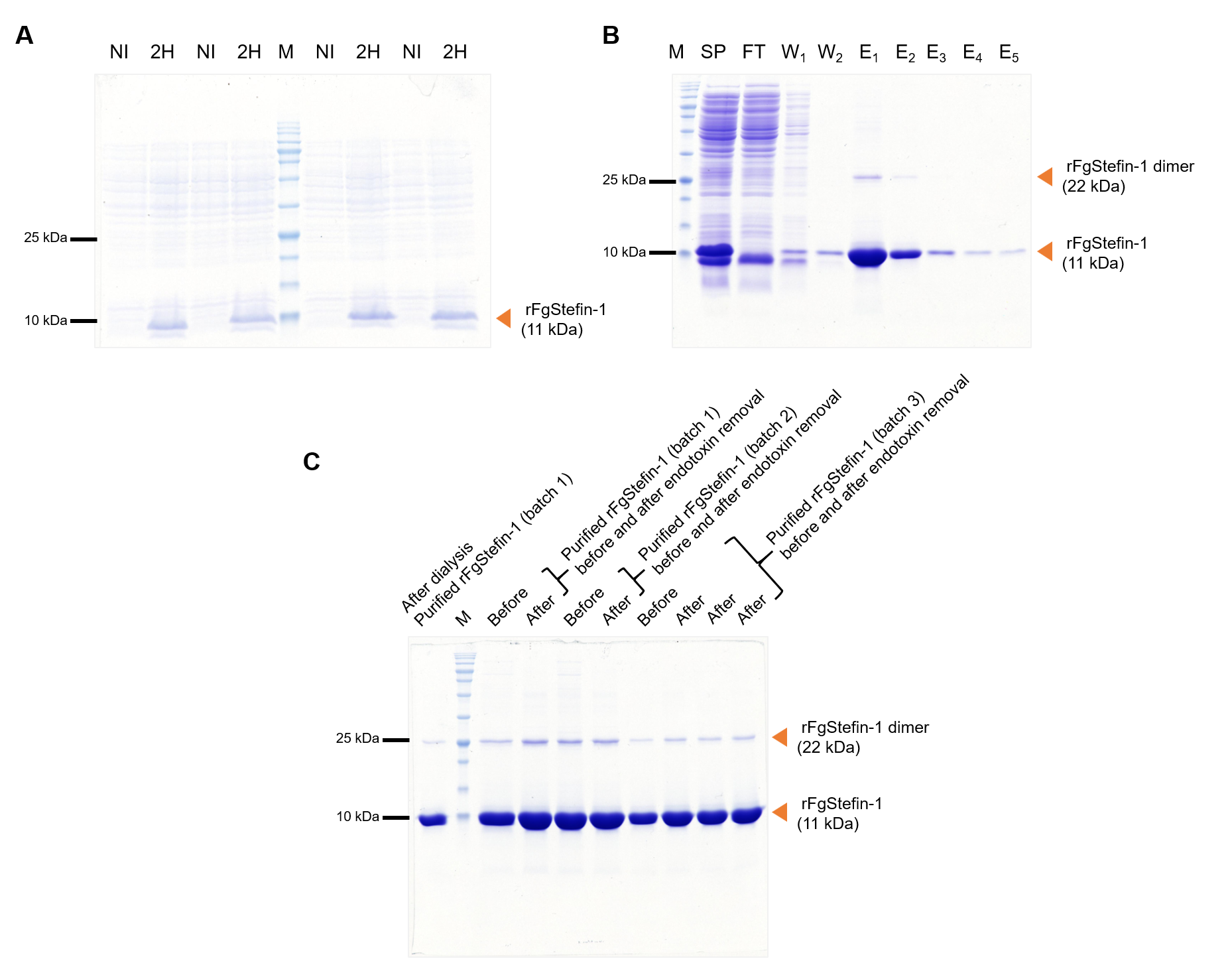

Supplement: S1 Fig — (A) Four clones of E. coli M15 containing pQE-30/FgStefin-1 were induced with 1 mM IPTG for 2 hours (2H), compared with non-induced (NI). (B) Purification of rFgStefin-1 by high-performance Ni Sepharose® (Cytiva, Uppsala, Sweden). using native conditions. (C) SDS-PAGE of purified rFgStefin-1 before and after endotoxin removal from the different batches. M: Whole Blue Range Prestained Protein Ladder (Vivantis, Malaysia); SP: Soluble protein fraction before purifying; FT: flow through; W1: wash fraction-1; W2: wash fraction-2; E1-E5: elution fraction 1–5. (TIF) [file pone.0353364.s001.tif]
