# Supplementary material for: Helminth-derived stefin-1 selectively reduces leukemic cell viability and promotes apoptosis in U937 cells
Source: PLoS One. 2026 Jul 27;21(7):e0353364. doi: 10.1371/journal.pone.0353364 (PMC13405116; doi:10.1371/journal.pone.0353364)

1st experiment

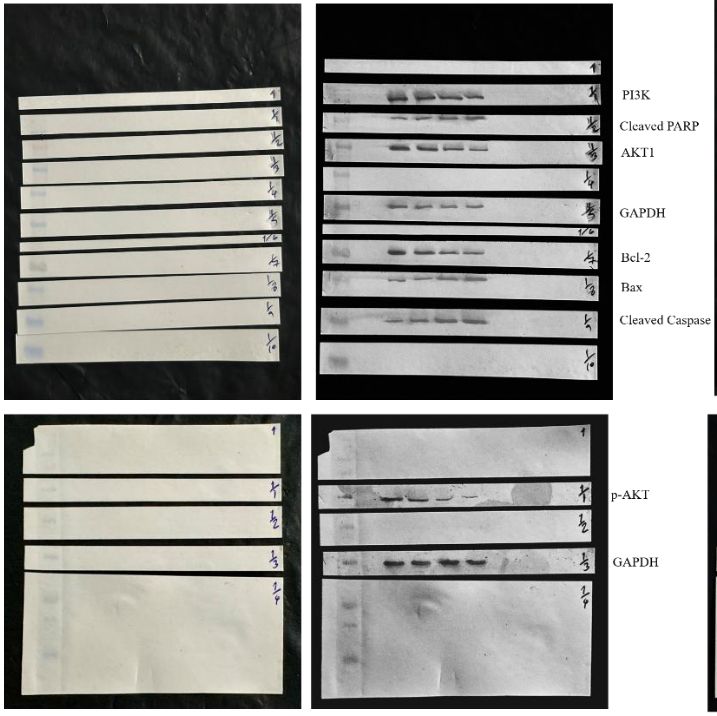

2nd experiment

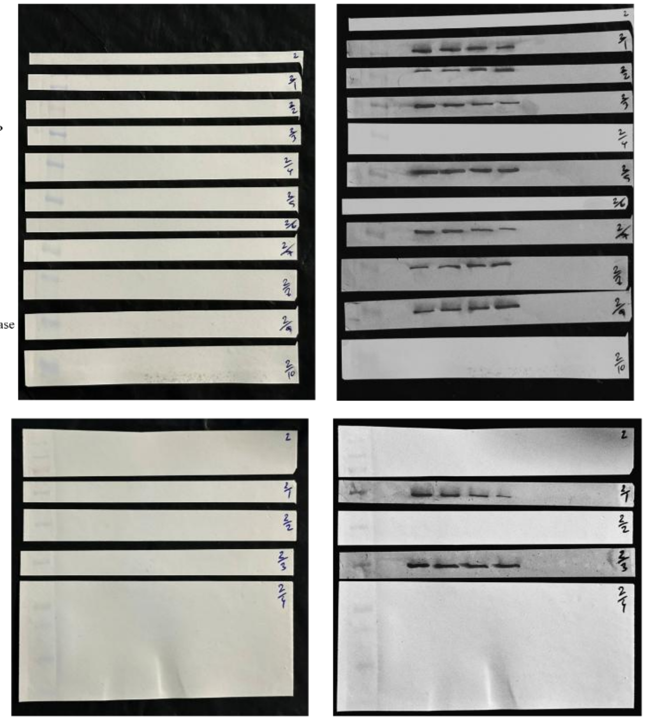

3rd experiment

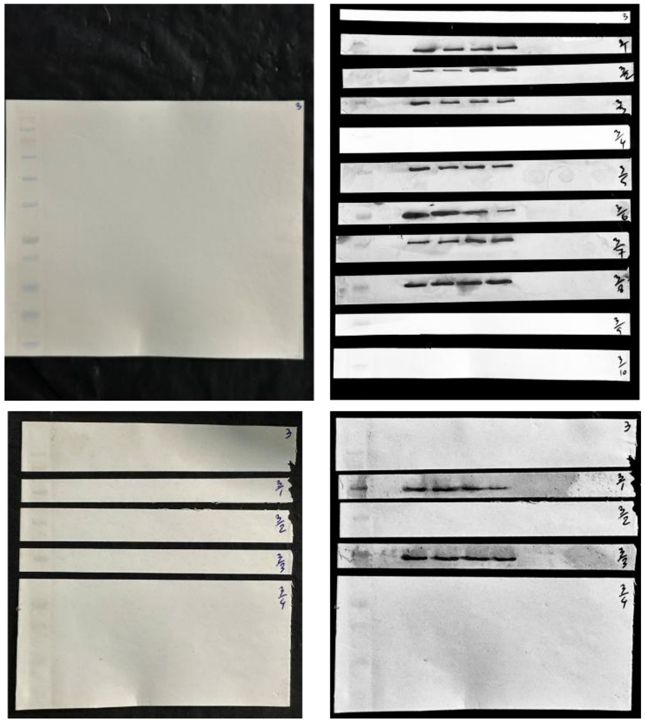

Supplement: S2 Fig — (PDF) [file pone.0353364.s002.pdf]
